# Vulvar cancer management and wrangling recurrent disease: A report from the society of gynecologic oncology journal club

**DOI:** 10.1016/j.gore.2023.101310

**Published:** 2023-11-28

**Authors:** Jessica E. Parker, Emi J. Yoshida, Lilian T. Gien, Brian M. Slomovitz, Christa Nagel

**Affiliations:** a Indiana University, Indianapolis, IN, USA; b University of California San Francisco, San Francisco, CA, USA; c University of Toronto, Toronto, ON, Canada; d Mount Sinai Medical Center, Miami Beach, FL, USA; e Ohio State University, Columbus, OH, USA

## Abstract

The Society of Gynecologic Oncology (SGO) Journal Club webinar series is an open forum that invites national experts to discuss the literature pertaining to important topics in the management of gynecologic cancers. On August 14th, 2023, SGO hosted a journal club focused on the management of upfront and recurrent vulvar cancer. Our discussants included Dr. Brian M Slomovitz from Mount Sinai Medical Center in Miami Beach, Dr. Emi Yoshida from the University of California San Francisco Helen Diller Family Comprehensive Cancer Center, and Dr. Lilian Gien from the University of Toronto Sunnybrook Odette Cancer Center. During the discussion,we reviewed the progression of vulvar cancer surgery from en bloc resection of the vulva and groins, to partial radical vulvectomy and sentinel lymph nodes. We also reviewed the management of node positive vulvar cancer including published and accruing Groningen International Study on Sentinel Nodes in Vulvar Cancer (GROINSS) trials and other sentinel trials from the Gynecologic Oncology Group (GOG). Here we will also review the literature on the management of recurrent vulvar cancer, highlighting current treatment options and ongoing clinical trials. The following is a report of the journal club presentation.

## Highlights

• Review the progression of vulvar cancer surgery from en bloc resection of the vulva and groins, to partial radical vulvectomy and sentinel lymph nodes.
• Examine the management of node positive vulvar cancer including published and accruing Groningen International Study on Sentinel Nodes in Vulvar Cancer (GROINSS) trials and other sentinel trials from the Gynecologic Oncology Group (GOG).
• Analyze literature on the management of recurrent vulvar cancer, highlighting current treatment options and ongoing clinical trials.

## 1 Historical data on the management of upfront vulvar cancer

Dr. Slomovitz began the journal club by reviewing historical data regarding surgery for vulvar cancer and prognostic indicators of recurrence. Historically, the surgical management of vulvar cancer included en bloc resection of the vulva with inguinofemoral lymphadenectomy (Way, 1948), which led to significant postoperative morbidity. Adoption of a modified radical vulvectomy with separate incision for lymphadenectomy allowed for reduced morbidity along with preservation of body image and sexual function. Moreover, the local recurrence rate for invasive disease did not differ between modified radical local excision versus the historic radical vulvectomy (DiSaia et al., 1979, Burke et al., 1995).

There have been several prognostic factors identified from the pathologic data of a radical vulvectomy that correlate to an increased risk of recurrence. One of the most important factors is margin status. Early data from 1990 showed a significant difference in the local recurrence rates between pathologically negative margins < 8 mm and >/= 8 mm (47.8 % vs 0 %) (Heaps et al., 1990). However, more recent data shows no significant difference in local recurrence rates between less than, and greater than, 8 mm margins (22.3 % vs 18.4 %, p = 0.09) (Groenen et al., 2010, Woelber et al., 2016, Arvas et al., 2018, Yang et al., 2020, Pleunis et al., 2018). Despite these data, the recommendation is still to aim for a 1–2 cm grossly negative surgical margin during radical vulvectomy, as one study showed that there was an increase in local recurrence rate with margin status of < 3 mm (Yang et al., 2020). Dr. Yoshida reviewed that HPV status and P53 mutational status also have prognostic implications and may eventually impact treatment decisions as the field progresses (Kortekaas et al., 2020).

Another factor which influences prognosis and recurrence is positive inguinofemoral lymph nodes. The results of GOG-36 showed that if suspicious or fixed lymph nodes were found clinically, the risk of additional groin node metastases was increased. This risk also increased with increasing age, tumor diameter, and tumor thickness (Homesley et al., 1993). Indications for inguinofemoral lymph node evaluation include tumor size greater than 2 cm, depth of invasion more than 1 mm, or presence of lymph vascular invasion if the previous two criteria were not met (Stehman and Look, 2006). Bilateral lymph node evaluation is indicated if the tumor is within 2 cm of the midline or on the anterior labia majora as the risk of bilateral lymph node metastasis is higher as the lesion approaches and crosses midline (Gonzalez Bosquet et al., 2007). Both progression-free and overall survival worsen significantly with inguinofemoral lymph node metastasis and therefore adjuvant radiotherapy is recommended (Mahner et al., 2015).

Dr. Yoshida discussed the role of radiation therapy in node positive vulvar cancer starting with GOG-37. This trial randomized patients with positive lymph nodes after radical vulvectomy and inguinofemoral lymphadenectomy to either pelvic radiotherapy to 45–50 Gy or pelvic lymphadenectomy, with the primary endpoint being overall survival. Overall survival was improved with radiotherapy in the subgroup of patients with two or more positive nodes or extra-nodal extension. Recurrence-free survival and cancer-related death were significantly improved with radiotherapy, highlighting the important role of radiation in the management of this disease (Kunos et al., 2009).

## 2 Sentinel lymphadenectomy

Dr. Slomovitz then discussed four trials pertaining to the concept of sentinel lymph node evaluation, which has transformed the standard of care in vulvar cancer. Groningen International Study on Sentinel Nodes in Vulvar Cancer (GROINSS)-VI was a multi-institutional clinical trial enrolling patients with squamous cell carcinoma of the vulva less than 4 cm in size. Participating centers were required to have verification of surgical competency in performing sentinel lymph node mapping. Patients on this trial underwent radical vulvectomy followed by inguinal sentinel lymph node evaluation with pathologic ultra staging (including routine hematoxylin and eosin staining followed by immunohistochemistry and further sectioning if routine staining is negative). Both lymphoscintigraphy and blue dye were used for mapping. If lymph nodes were negative, no further treatment was performed. If positive, the patients underwent full inguinofemoral lymphadenectomy, and if additional nodal metastasis were found, the patient received radiation to the groin and pelvis. Sentinel node dissection, in comparison with full inguinofemoral lymphadenectomy, was found to have significantly less morbidity with decreased wound breakdown, infection, hospital stay, and lymphedema. In patients with negative sentinel lymph nodes, the 2-year recurrence rate to the groin was 3 % overall, 2.3 % for unifocal disease (Van der Zee et al., 2008). The long-term follow-up of this study was published in 2016 and showed significantly worse disease-specific survival with positive sentinel lymph nodes (Te Grootenhuis et al., 2016).

GOG-173 was also a multi-institutional trial of patients with squamous cell carcinoma of the vulva 2–6 cm in size evaluating the safety of using sentinel lymph node biopsy to replace inguinofemoral lymphadenectomy. As opposed to the GROINSS-VI trial, there was no skill verification required, all patients had blue dye but not all had lymphoscintigraphy, and all patients underwent both sentinel lymph node dissection followed by full inguinofemoral lymphadenectomy at the time of surgery. The primary endpoint was accuracy of sentinel evaluation determined by the false negative predictive value. It was found that in 55 % of node-positive cases, the sentinel node was the only positive lymph node and that the false negative predictive value was 4 %. The false negative predictive value for tumors less than 4 cm was 2 % (Levenback et al., 2012). Therefore, these GROINSS-VI and GOG-173 conclude that sentinel lymph node evaluation is feasible, decreases morbidity, and is therefore recommended in patients with tumors less than 4 cm.

In the second GROINSS study, GROINSS-VII, patients with unifocal vulvar squamous cell carcinoma less than 4 cm were enrolled if they had clinically negative nodes and preoperative imaging showing lymph nodes < 1.5 cm. If sentinel nodes were negative, they were observed. If positive, they received radiation to a total dose of 50 Gy. The choice of the addition of chemotherapy with cisplatin at the time of radiotherapy was left to the discretion of the participating institution. The goal of the study was to determine if radical surgery could be avoided in patients with positive lymph nodes. However, upon interim analysis it was found that patients with sentinel lymph node metastasis > 2 mm had a 20 % groin recurrence rate, and thus the study protocol was altered so that this subset of patients received complete inguinofemoral lymphadenectomy prior to receiving radiation. Results showed that the 2-year groin recurrence rate in patients with micro metastases (<=2mm) was 1.6 % if radiation was given. If patients did not receive radiation, the recurrence rate was as high as 11.8 %. Alternatively, in those with metastases > 2 mm, the addition of inguinofemoral lymphadenectomy to radiation decreased the 2-year groin recurrence rate significantly from 22 % to 6.9 %. This study concluded that complete inguinofemoral lymphadenectomy could only be omitted in the setting of micro metastatic disease. Unfortunately, the rate of lymphedema was 32 % in the patients who underwent full inguinofemoral lymphadenectomy as opposed to a 16.4 % in patients who had radiotherapy alone (Oonk et al., 2021).

GROINNS-VIII (NCT05076942) is a study that is currently ongoing. The rationale for this study is to attempt to decrease surgical morbidity of inguinofemoral lymphadenectomy in patients with > 2 mm lymph node metastases by improving efficacy of radiotherapy. The study aims to do this by increasing the radiotherapy dose from 50 to 56 Gy and giving concurrent chemotherapy with weekly cisplatin as is done in both cervical and locally advanced vulvar cancer. The primary objective is to evaluate the safety of replacing lymphadenectomy with chemoradiation for patients with early-stage vulvar cancer and macro metastatic (>2mm) lymph node involvement. Quality of life is also being evaluated.

In summary, standard of care for early-stage invasive vulvar cancer is radical excision of the primary tumor with sentinel groin lymph node dissection. If the sentinel node is negative, surveillance is recommended. For lymph node metastases < 2 mm, adjuvant treatment with groin radiation to 50 Gy without lymphadenectomy is safe. With larger metastases, inguinofemoral lymphadenectomy prior to groin radiation is currently recommended to reduce the risk of groin recurrence, although our preference would be to enroll the patient on GROINNS-VIII.

The speakers were then asked questions from the audience. Dr. Gien and Dr. Slomovitz discussed the use of indocyanine green (ICG) for sentinel lymph node mapping, keeping in mind limitations of using ICG alone with spillage of the dye, or difficulty locating the sentinel node without an auditory cue, especially in more obese patients. They reminded the audience that patients with suspicious nodes on preoperative imaging were not eligible for any of the GROINSS studies and thus debulking the groin nodes in these circumstances should be considered. The speakers also emphasized the difficulty in performing clinical trials in this rare cancer type and alluded to possible future trials on the role of neoadjuvant chemotherapy as well as immunotherapy in vulvar cancer.

## 3 Locally advanced vulvar cancer

Dr. Yoshida then presented two sentinel trials on the role of chemoradiation in the management of locally advanced vulvar cancer. GOG-205 was reviewed, which examined the feasibility of surgery after chemoradiation with cisplatin in patients with T3/T4 tumors unresectable by radical vulvectomy. These patients received a radiotherapy treatment dose of 57.6 Gy. Pathologic complete response was found in approximately 50 % of the total population. Among patients with a complete clinical response who underwent surgical biopsy, 78 % were also found to have a complete pathologic response. Toxicity was felt to be acceptable in this population, and therefore neoadjuvant chemoradiation remains a treatment option for the appropriate patient (Moore et al., 2012).

GOG-279 was presented as a plenary session at the 2023 SGO annual meeting. This study evaluated patients with T2 or T3 squamous cell carcinoma of the vulva, not amenable to standard radical vulvectomy, and stratified them based on resectable versus unresectable lymph nodes and node positivity. In this study, the vulva was given an increased dose of radiation to 64 Gy and gemcitabine was added to cisplatin weekly throughout radiation. The pathologic complete response rate in this cohort was 73.1 %, however there were significant toxicities, with 25 patients having grade 3 adverse events, 18 grade 4, and one grade 5. Progression free survival was 74 % at 12 months and overall survival at 24 months was 69.6 % (Horowitz et al., 2023).

Dr. Yoshida then presented a clinical case of a patient with squamous cell carcinoma of the vulva and discussed radiation treatment planning. The video is available within the journal club presentation. She also reviewed the radiation doses commonly used, highlighting that 50–56 Gy is used for microscopic disease and 60–66 Gy is used for definitive treatment.

## 4 Management of recurrent or metastatic vulvar cancer

Dr. Gien then began her presentation on the management of recurrent or metastatic vulvar cancer. Once squamous cell carcinoma of the vulva has metastasized or recurred, guidelines on how to treat these patients are based on scarce data with small populations, variability of inclusion criteria, and lack of large prospective multicenter randomized controlled trials. Furthermore, there has been no improvement in survival in these populations over the last two decades.

In patients with distant recurrences, there is a 5-year survival of < 10 % (Zach et al., 2021). Much of the data on treatment is extrapolated from cervical cancer or is anecdotal in nature. Cisplatin was first used for treatment of these patients, however single-agent cisplatin administered every 21 days showed a 0 % response rate, thought to be due to recurrence within an irradiated field (Thigpen et al., 1986). The use of single-agent paclitaxel showed a low response rate of 13.8 %. It is important to take into consideration that 69 % of these patients had previous radiation (Witteveen et al., 2009). When doublet therapy was introduced with cisplatin and vinorelbine, one study showed a response rate of 40 %. However, this study was small with a sample size of 16 patients and excluded those with prior radiation (Cormio et al., 2009).

Targeted therapies, such as anti-angiogenic treatments, are often used in cervical cancer but have not been studied in vulvar cancer. There have been case series presented, such as one study examining the use of bevacizumab concomitantly with platinum-based chemotherapy followed by maintenance bevacizumab in patients with vulvar cancer. Out of the nine patients in this series, there was a complete response in two patients, partial response in one, and stable disease in three (Woelber et al., 2021). Other targeted therapies such as EGFR inhibitors may have a role in the treatment of vulvar cancer as high levels of EGFR amplification are linked to poor overall survival (Growdon et al., 2008). One phase two trial evaluated erlotinib in 41 patients with locally advanced, metastatic, or recurrent vulvar cancer, and found an overall clinical benefit rate of 67.5 %. However, the responses were short in duration with progression by 3 months and significant toxicity (Horowitz et al., 2012). Additional case series or reports on the use of erlotinib show variable outcomes (Woelber et al., 2021, Olawaiye et al., 2007, Inrhaoun et al., 2012).

There have also been several case series on the use of immunotherapy in vulvar cancer. Immunotherapy has been hypothesized to have activity in vulvar cancer since up to 73 % express PDL-1 with moderate-strong expression in 27 % (Choschzick et al., 2018). KEYNOTE-158 evaluated 101 patients with previously treated metastatic or unresectable vulvar squamous cell carcinoma who were given pembrolizumab every 3 weeks. Of note, 83 % had PDL-1 positive tumors. The overall response rate was only 10.9 %, but duration of response was over 20 months, and patients with PDL-1 negative tumors responded similarly to those with PDL-1 positive tumors suggesting that PDL-1 status may not predict response in these patients (Shapira-Frommer et al., 2022). Checkpoint inhibitors, specifically nivolumab, have also been studied in a limited capacity. The Checkmate-358 trial included three vulvar and two vaginal cancer patients. Of these five patients, there was only one partial response (Naumann et al., 2019). Further studies could consider the role of combination therapies with consideration of immunotherapy, as is being studied in cervical cancers.

Dr. Gien then summarized the guidelines for treatment of advanced or recurrent/metastatic vulvar cancer. Preferred regimens are single agent platinum or platinum doublets with consideration of bevacizumab. Other recommended agents to be considered are paclitaxel, erlotinib, pembrolizumab, or nivolumab. The strongest recommendation is for enrollment in a clinical trial if available. Further studies are needed but randomized controlled trials are not feasible.

## 5 Conclusions

Significant strides have been made in the treatment of upfront vulvar cancer, and future studies are focusing on further improving clinical outcomes while minimizing toxicities, especially with the adoption of sentinel lymph node mapping. However, there is still much to be improved upon in the management of recurrent or metastatic disease. The ability to perform clinical trials in vulvar cancer is limited by the rarity of this tumor type. We will continue to push forward to wrangle this difficult disease.

**Author contributions**

Drs. Parker and Nagel developed the clinical commentary. Drs. Slomovitz, Yoshida and Gien reviewed and provided edits.

## Declaration of competing interest

The authors declare that they have no known competing financial interests or personal relationships that could have appeared to influence the work reported in this paper.
